# Predictors of Neurological Outcome Following Subaxial Cervical Spine Trauma

**DOI:** 10.7759/cureus.6402

**Published:** 2019-12-17

**Authors:** Frederick L Hitti, Brendan J Mcshane, Andrew I Yang, Cole Rinehart, Ahmed Albayar, Marc Branche, Yagiz U Yolcu, Zarina S Ali, James M Schuster, Ali K Ozturk

**Affiliations:** 1 Neurosurgery, University of Pennsylvania, Philadelphia, USA

**Keywords:** subaxial cervical spine, trauma, asia, outcomes, predictors, slic

## Abstract

Background

The treatment of traumatic subaxial cervical spine injuries remains controversial. The American Spinal Injury Association (ASIA) impairment scale (AIS) is a widely-used metric to score neurological function after spinal cord injury (SCI). Here, we evaluated the outcomes of patients who underwent treatment of subaxial cervical spine injuries to identify predictors of neurologic function after injury and treatment.

Methods

We performed a retrospective logistic regression analysis to determine predictors of neurological outcome; 76 patients met the inclusion criteria and presented for a three-month follow-up. The mean age was 50.6±18.7 years old and the majority of patients were male (n=49, 64%).

Results

The majority of patients had stable AIS scores at three months (n=56, 74%). A subset of patients showed improvement at three months (n=16, 21%), while a small subset of patients had neurological decline at three months (n=4, 5%). In our model, increasing patient age (odds ratio [OR] 1.39, 1.10-2.61 95% confidence interval [CI], P<0.001) and a previous or current diagnosis of cancer (OR 22.4, 1.25-820 95% CI, P=0.04) significantly increased the odds of neurological decline at three months. In patients treated surgically, we found that delay in surgical treatment (>24 hours) was associated with a decreased odds of neurological improvement (OR 0.24, 0.05-0.99 95% CI, P=0.048). Cervical spine injuries are heterogeneous and difficult to manage.

Conclusion

We found that increasing patient age and an oncologic history were associated with neurological deterioration while a delay in surgical treatment was associated with decreased odds of improvement. These predictors of outcome may be used to guide prognosis and treatment decisions.

## Introduction

The incidence of cervical spine trauma, which accounts for nearly half of all spinal cord injuries (SCIs) in North America, is increasing [1-2]. Injuries to the cervical spine can lead to devastating neurologic impairment [3]. While several classification systems exist, subaxial spine injuries are broadly defined as fractures, dislocations, or ligamentous disruption of the C3 to C7 vertebral levels. Many of these injuries can lead to cervical spine instability and SCI [4-5]. Proper diagnosis and treatment of these injuries is essential.

Injury to the subaxial cervical spine may be managed expectantly for stable injuries whereas unstable injuries may be treated with operative or non-operative stabilization [6]. If decompression of the spinal cord is necessary, surgical management must be pursued. Non-surgical stabilization may be achieved with a cervical collar or halo. Surgical stabilization may be achieved via anterior, posterior, or combined approaches in which hardware and bone graft are placed to stabilize and fuse the cervical spine. Furthermore, dislocations may be reduced by closed or open approaches [7].

Due to the complex structure of the cervical spine, cervical spine trauma can produce heterogeneous patterns of injury. Classifications systems such as the subaxial cervical injury classification (SLIC) and the AOSpine subaxial cervical injury classification systems have been developed to aid in diagnosis and treatment [1,4-5]. The SLIC system provides a score that guides surgical versus non-surgical treatment, while the AOSpine classification system provides a diagnostic tool to standardize descriptions of injury patterns [4-5].

While the classification systems described above can be used to guide diagnosis and treatment, there have been few studies examining the injury, patient, or treatment-specific factors that can predict neurological outcome following injury and treatment [8-9]. One prior study found that prognosis was poor in patients with spinal stenosis, soft tissue damage, or neurological impairment [8]. We aimed to examine other patient and treatment factors that could help guide prognosis following cervical spine injury. We hypothesized that recovery following SCI could be improved if modifiable prognostic indicators were discovered.

## Materials and methods

Patient selection

We obtained approval from our institutional review board (IRB) to retrospectively identify all consecutive patients at our institution who presented with acute non-penetrating subaxial cervical spine injury from 2007 to 2016. This patient population included patients with fractures or dislocations of C3-C7.

Clinical data collection

Once the target patient population was identified, we collected relevant data from medical records- both paper and electronic (Epic, Epic Systems Corporation, Madison, Wisconsin). Demographic information was collected and medical comorbidities at the time of initial injury were noted. SLIC scores at presentation were calculated retrospectively by the authors (FLH and CR). In addition, American Spinal Injury Association (ASIA) impairment scale (AIS) scores at the time of initial presentation and at three-month follow-up were collected to assess neurological function before and after the subaxial cervical spine injury [10]. Patients who did not follow-up at three months were excluded from the study. We also recorded the time to neurosurgical evaluation from the initial injury, length of hospitalization, and time to surgery if surgery was performed.

Statistical analysis

Statistical analysis was performed using Microsoft Excel and R. Logistic regression analysis was performed using the generalized linear model in R. Type II analysis of variance (ANOVA) was then utilized to compute likelihood ratios and P values. Results were considered significant if P < 0.05. Data are presented as mean ± standard deviation (SD). Two-tailed student’s t-test and Fisher's exact test were used to compare the groups where appropriate.

## Results

Study population

**Figure 1:**
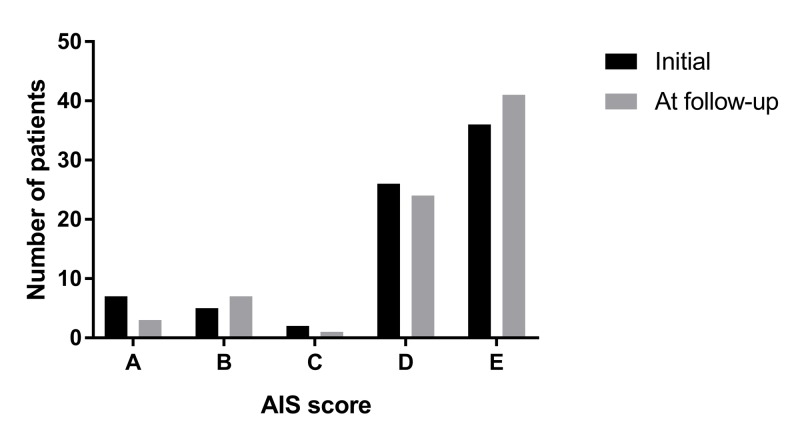
AIS scores at presentation and follow-up AIS: American Spinal Injury Association impairment scale

We initially identified 109 patients that presented to our institution following acute non-penetrating subaxial spine trauma. Of these patients, 76 (70%) presented for their three-month follow-up. In this study, we examined the neurological outcomes of this subgroup of patients for which follow-up was available. The demographics of this cohort of patients is shown in Table 1. The mean age was 50.6 ± 18.7 years old and the majority of patients were male (n = 49, 64%). With regards to race, the majority of patients were Caucasian (n = 37, 49%). A large percentage of the patients were African American (n = 29, 38%). We tracked patient comorbidities and found diabetes, cancer, osteoporosis, coronary artery disease (CAD), and peripheral arterial disease (PAD) in 11%, 11%, 8%, 8%, and 7% of patients respectively. The majority of patients (n = 48, 63%) were surgically stabilized with anterior (n = 15), posterior (n= 19), or anterior and posterior (n = 14) cervical fixation. The remainder of the patients (n = 28, 37%) were stabilized non-operatively with a cervical collar. The mean SLIC score of the patients that underwent surgery was 6.7 ± 1.9; it was 3.6 ± 1.5 in the non-operative group. The distribution of patients’ initial and follow-up AIS scores is shown in Figure 1 and in Table 2. Regarding patient outcomes, the majority of patients had a stable AIS score at three months (n = 56, 74%). A subset of patients did demonstrate improvement at three months (n = 16, 21%). Unfortunately, a small subset of patients had neurological decline at three months (n = 4, 5%).

**Table 1:** Demographics and outcomes AIS: American Spinal Injury Association impairment scale; CAD: coronary artery disease; PAD: peripheral arterial disease; SLIC: subaxial cervical injury classification

Demographic	
Age	50.6 ± 18.7 (range 18-90, median 47.5)
Sex	Male 49 (64%), Female 27 (36%)
Race	Caucasian 37 (49%), African American 29 (38%), Asian 4 (5%), Other 6 (8%)
Comorbidities	Osteoporosis 6 (8%), Diabetes 8 (11%), CAD 6 (8%), PAD 5 (7%), Cancer 8 (11%)
Management	Operative 48 (63%), Non-operative 28 (37%)
SLIC scores – Operative group	6.7 ± 1.9 (range 3-9, median 7)
SLIC scores – Non-operative group	3.6 ± 1.5 (range 2-7, median 4)
AIS score at three months	Stable 56 (74%), Improved 16 (21%), Worse 4 (5%)

**Table 2:** AIS scores at presentation and follow-up AIS: American Spinal Injury Association (ASIA) impairment scale

AIS score	Initial score – all patients	At follow-up – all patients	Initial score – surgical patients	At follow-up – surgical patients	Initial score – non-operative patients	At follow-up – non-operative patients
A	7	3	7	3	0	0
B	5	7	5	7	0	0
C	2	1	1	1	1	0
D	26	24	20	19	6	5
E	36	41	15	18	21	23

Effect of demographics on neurological outcome

We hypothesized that patient demographics may predict neurological outcome following subaxial cervical spine trauma. To assess this, we performed logistic regression analysis followed by type II ANOVA. While patient age, race, or sex did not predict odds of improvement in AIS score at three months, patient age was significantly associated with a decrement in AIS score at three months (Table 3). In this model, increasing patient age increased the odds of neurological decline, odds ratio (OR) 1.39 (1.10 - 2.61, 95% confidence interval [CI]), P = 0.00004. Patients who initially presented neurologically intact (AIS E) were also compared to patients who presented with neurological injury (AIS A, B, C, or D). Univariate logistic regression did not reveal a significant difference between these groups on odds of neurological decline (P = 0.25).

**Table 3:** The effect of demographics on odds of AIS score worsening at three-month follow-up AIS: American Spinal Injury Association (ASIA) impairment scale; CI: confidence interval; LR: likelihood ratio; OR: odds ratio; P: probability

Demographic	OR	95% CI	LR	P value
Age	1.39	1.10 – 2.61	16.7	0.00004
Race	1.69	0.47 – 10.1	0.55	0.46
Sex	5.42	0.35 – 277.5	1.23	0.27

Effect of patient comorbidities on neurological outcome

In addition to patient age, sex and race, we investigated whether comorbid medical conditions could influence neurological outcomes following subaxial cervical spine trauma. We examined the effect of osteoporosis, diabetes, CAD, PAD, and cancer on neurological outcomes at three months using logistic regression followed by type II ANOVA (Table 4). While none of these conditions was associated with improvement in AIS score at three months, carrying a previous cancer diagnosis was associated with neurological deterioration at three months. A history of cancer significantly increased the odds of AIS score decrement, OR 22.4 (1.25 - 820, 95% CI), P = 0.04.

**Table 4:** The effect of comorbidities on odds of AIS score worsening at three-month follow-up AIS: American Spinal Injury Association (ASIA) impairment scale; CAD: coronary artery disease; CI: confidence interval; LR: likelihood ratio; OR: odds ratio; P: probability; PAD: peripheral arterial disease

Comorbidity	OR	95% CI	LR	P value
Osteoporosis	1.98	0.01 – 241	0.08	0.78
Diabetes	0.79	0.01 – 69.7	0.01	0.92
CAD	10.7	0.26 – 573	1.69	0.19
PAD	8.16	0.26 – 501	1.44	0.23
Cancer	22.4	1.25 – 820	4.42	0.04

Effect of delay in surgical stabilization on neurological outcome

We hypothesized that delay in surgical stabilization would have a negative impact on neurological recovery. To examine this, we noted the time to surgery from initial trauma evaluation. In the patients that underwent surgical stabilization, 50% (n = 24) were surgically stabilized 24 hours after initial evaluation. Delays in surgery could be due to a variety of factors that could confound this analysis. To attempt to control for this, we considered delays in surgery in the context of length of hospital stay since medically complex patients tend to have longer hospitalizations. The average length of the acute hospitalization was 14.1 ± 10.9 days in the cohort of patients undergoing surgery. We performed logistic regression followed by type II ANOVA to examine the effect of delay in surgery and length of hospitalization on odds of improvement in neurological outcomes. We found that delay in surgical treatment was associated with a decreased odds of neurological improvement, OR 0.24 (0.05 - 0.99, 95% CI), P = 0.048. Length of hospitalization was not significantly associated with neurological improvement at three months (Table 5). The mean age of the patients who underwent surgery in a timely fashion was not significantly different than the mean age of the patients who underwent surgery in a delayed fashion (46.7 ± 15.6 years old vs. 53.1 ± 19.6 years old, p = 0.228). The sex of the patients in the two groups was not significantly different either (66.7% male in the timely group vs. 75% male in the delayed group, p = 0.7516). In both groups, nine patients underwent attempted closed reduction with cervical traction pre-operatively (p > 0.9999). The majority of patients in both groups were unable to be reduced with traction, and there was no significant difference between the groups (77.8% were unable to be reduced in the timely group vs. 66.7% were unable to be reduced in the delayed group, p > 0.9999). We also compared SLIC scores at presentation between the two groups. Interestingly, there was a significant difference in pre-operative SLIC scores between the two groups (7.42 ± 1.58 in the timely group vs. 6.08 ± 1.80 in the delayed group, p = 0.010).

**Table 5:** The effect of time to surgery and length of hospitalization on odds of AIS score improvement at three-month follow-up AIS: American Spinal Injury Association (ASIA) impairment scale; CI: confidence interval; LR: likelihood ratio; OR: odds ratio; P: probability

	OR	95% CI	LR	P value
> 24 hours from injury to surgery	0.24	0.05 – 0.99	3.90	0.048
Length of hospitalization	1.00	0.93 – 1.06	0.01	0.929

## Discussion

Cervical spine trauma is a frequent cause of SCI and can result in severe neurological dysfunction [1-2]. As the structure and function of C1 and C2 vary considerably from the subaxial spine (C3 to C7), these anatomical groups are considered separately when diagnosing, treating, and investigating cervical spine injury. In the present study, we aimed to examine prognostic indicators of neurological function after subaxial spine injury using the AIS scoring system as a metric of neurological function [10].

The two most popular classification systems for cervical spine injury are the AOspine classification and SLIC systems [1,4-5]. These systems provide a means to classify injuries into diagnostic categories based on injury pattern (AOspine) and also allow for guidance regarding operative versus non-operative management (SLIC). These classification paradigms are somewhat limited in their ability to provide prognostic information regarding neurological recovery following injury and subsequent treatment [9].

We first examined patient demographic factors to determine if age, race, sex, or medical comorbidities were associated with neurological recovery or decline. We found that race and sex were not associated with neurological outcome following injury and treatment. Interestingly, we found that increasing age was associated with increased odds of neurological deterioration. One prior study, however, found that neither sex nor age was associated with neurological outcome following subaxial spine injury [8]. There are several important differences between the present study and the previous one. The previous study only included patients who were managed operatively. Furthermore, the previous study divided patients into two groups. One group was 65 years of age or greater and the other group was younger than 65 years old. In the present study, we included both surgical and non-surgical patients. Additionally, we analyzed the effect of age as a continuous variable instead of categorizing age into two groups. These differences in study design may account for the differences in our findings. 

With regard to comorbidities, we found that patients with any cancer diagnosis had increased odds of neurological deterioration after subaxial cervical spine injury. The mechanism underlying this association remains to be elucidated. Neurological recovery could be impaired by a history of cancer treatment or possibly by an inflammatory state hostile to recovery. Indeed, many traditional cancer treatments and newer targeted or immunomodulatory agents have known neurological side effects [11]. It is interesting to speculate that these therapies could have long-lasting effects on neurological recovery even when discontinued. Our data suggest that patients with a cancer diagnosis should be counseled regarding the risk of neurological deterioration following cervical spine injury.

It is not readily apparent why some patients would undergo neurological decline after their initial presentation. While two patients in our series underwent re-operation for hardware failure/loss of alignment, neither of these patients demonstrated neurological decline. Additionally, we did not observe any post-operative hematomas that could explain the decline. These data argue against iatrogenic causes of decline. The cause for neurological decline is possibly related to progressive edema or ischemia of neural elements, however, the data to examine these hypotheses definitively is not available.

Patient demographics are not modifiable factors, so the aforementioned findings will mainly serve to inform patient counseling. We sought to also examine whether modifiable factors, such as time to surgery, would affect neurological recovery after subaxial cervical spine injury. Interestingly, we found that delay in time to surgery of greater than 24 hours after injury was associated with decreased odds of neurological recovery. There can be many reasons for delayed surgical treatment including delay in presentation, delay in diagnosis, delay in evaluation, and delay in treatment. It is important that healthcare systems strive to improve the timeliness of treatment.

The most parsimonious explanation for our findings is that neurological recovery is enhanced by more rapid treatment of neurologic compression. Indeed, other patient factors such as age, sex, or history of attempted closed reduction with cervical traction could affect neurological recovery. We did not find a significant difference, however, between the delayed operative and timely operative groups in these domains. The success of closed reduction was also not significantly different between the two groups. Interestingly, the groups did demonstrate a significant difference in SLIC score at presentation. The patients that underwent surgery sooner had a greater SLIC scores compared to the patients that underwent surgery in a delayed fashion. This may reflect a trend to operate more expediently in patients with a greater severity of injury. While statistically significant, the absolute difference between the mean SLIC scores (1.34 points) was not great. Furthermore, the delayed group had a decreased odds of improvement despite a less severe injury as measured by the SLIC score. We would expect patients with a less severe injury to exhibit a greater likelihood of recovery. For the aforementioned reasons, we believe that the delay in time to surgery most likely explains the decreased odds of improvement in this group of patients.

There has been some debate regarding the timing of operative treatment of cervical spine injury [12-16]. There is a growing consensus that operative intervention, if indicated, should be done sooner rather than later (less than 24 hours after initial injury). There is also evidence to suggest that earlier intervention results in reduced cost [17]. The literature is not definitive regarding clinical outcomes, however, the Surgical Timing in Acute Spinal Cord Injury Study (STASCIS) has provided excellent evidence that earlier intervention improves the likelihood of improved neurological recovery in patients with SCI [13]. These investigators found that an increased number of patients had an improvement of two or three AIS grades in the early intervention group versus the late intervention group. We found that earlier intervention improved the odds of any neurological recovery on the AIS scale. Together, these findings provide strong support for earlier intervention. Furthermore, our study includes analysis of SLIC scores and supports the utility of this clinical scale.

We must acknowledge some limitations of our study. Other than the inherent limitations of a retrospective review, our study included a relatively small number of patients from a single center. Hence, it is subject to possible sampling bias and may lack generalizability. Additionally, the majority of our patients had stable neurological outcomes after injury. While treatment may have prevented neurological decline that may have occurred without treatment, we cannot determine that from this dataset. We also may have observed stable neurological outcomes in many of our patients due to the limited gradations of the AIS scale. A more granular outcome score may have allowed us to detect more subtle outcome changes and possibly a greater number of prognostic indicators. With these limitations in mind, we believe that our results merit consideration and further study.

## Conclusions

Cervical spine injury diagnosis and treatment is difficult given the wide range of injury patterns and neurological effects. While several classification systems exist, prognostic indicators of neurological recovery after injury and treatment are limited. We found that increasing patient age and an oncologic history were associated with neurological deterioration. While these are not modifiable factors, these findings may help guide discussions with patients regarding prognosis. Interestingly, we found that delay in surgical treatment was associated with decreased odds of neurological improvement. These results suggest that if surgical treatment is indicated, it should be done within 24 hours of injury.
